# EBV-Positive Pleomorphic Variant Transformation of CD5-Negative Mantle Cell Lymphoma: A Rare Case Report and Literature Review

**DOI:** 10.1155/2024/3267739

**Published:** 2024-06-05

**Authors:** Amy Song, Julie Y. Li, Samuel G. Cockey, Richard Shao, Hailing Zhang

**Affiliations:** ^1^Department of Internal Medicine, Columbia University Irving Medical Center, New York, NY 100032, USA; ^2^Department of Hematopathology and Lab Medicines, H. Lee Moffitt Cancer Center and Research Institute, 12902 USF Magnolia Drive, Tampa, Florida 33612, USA; ^3^Morsani College of Medicine, University of South Florida Health, Tampa, FL 33602, USA; ^4^The University of Central Florida College of Medicine, 6850 Lake Nona Blvd, Orlando, FL 32827, USA

## Abstract

Mantle cell lymphoma (MCL) is a mature B-cell lymphoma associated with cyclin D family rearrangements and typically expresses CD5 and cyclin D1. Epstein–Barr virus- (EBV-) positive MCL is rare, and the role of EBV infection and its transformation in MCL remains unclear. We present a case of CD5-negative classic MCL that progressed to an EBV + pleomorphic MCL six years after the initial diagnosis. Molecular studies confirmed the same clonal origin. To the best of our knowledge, the EBV-positive transformation of CD5-negative MCL into a pleomorphic variant has rarely been reported, and its recognition is important for the diagnosis and the management of patients with MCL.

## 1. Introduction

Mantle cell lymphoma (MCL) is a B-cell non-Hodgkin's lymphoma characterized by the genetic hallmark translocation of t (11; 14) (q13; q32) IgH::CCND1 that affects more than 95% of the patients [1]. The majority of the patients present with lymphadenopathy and frequent involvement of the bone marrow, spleen, peripheral blood, and gastrointestinal tract. Despite being considered incurable, the median overall survival has significantly improved due to advancements in therapies.

Classic or typical MCL features a monomorphic small to medium sized lymphoid cells. These cells exhibit irregular nuclear contours, limited cytoplasm, condensed chromatin, and inconspicuous nucleoli, resembling centrocytes [1]. However, a diverse cytological variants has been recognized, including small cell, blastoid, pleomorphic, and marginal zone-like subtypes [1], posing challenges for diagnosis. The small cell variant resembles chronic lymphocytic leukemia. Pleomorphic and blastoid variants are considered high-risk MCLs, and they are often associated with an elevated Ki-67 proliferation index (>30%) and a less favorable prognosis [1]. The blastoid type resembles lymphoblasts, while the pleomorphic type displays large cells with irregular nuclei, prominent nucleoli, and pale cytoplasm reminiscent of diffuse large B-cell lymphoma [2]. MCLs with classic and small cell morphologies may progress to pleomorphic or blastoid morphologies, although an inverse evolution pattern has rarely been reported [3].

MCL cells express pan B-cell markers (CD19, CD20, CD22, CD79a, and PAX5), and are usually characterized by the co-expression of CD5 and cyclin D1. However, approximately 5% of MCLs are CD5-negative, presenting similarly to their CD5+ counterparts but showing a relatively better prognosis than CD5+ MCLs [4].

EBV is an oncogenic virus linked to various B-lymphoid malignancies, including Burkitt lymphoma, diffuse large B-cell lymphoma, and Hodgkin lymphoma [5]. The association between EBV and MCL has rarely been reported, with an estimated incidence of approximately 5.8% of reported MCL cases being EBV-positive. EBV + cells in MCL varied in number, and interestingly, most of the EBV + cells are background CD3+ T cells, instead of tumor cells [6]. Furthermore, the EBV-associated transformation of MCL has rarely been reported [7], and the role of EBV in MCL transformation remains unclear. Here, we present a case in which a EBV-negative, CD5-negative classical MCL progressed to EBV + pleomorphic variant.

## 2. Case Presentation

This is a 67-year-old male who had a history of low-grade CD5-negative mantle cell lymphoma diagnosed at outside hospital in 2017 through cecal mass biopsy with bone marrow and spleen involvement. The outside bone marrow biopsy reported the lymphoma cells to be positive for cyclin D1 and negative for CD5 and SOX11 by immunohistochemical stains. He was treated with 6 cycles of bendamustine and rituximab from September 2017 to March 2018. He had a good response to therapy with a decrease in the size of the spleen and no other organomegaly or lymphadenopathy.

**Figure 1:**
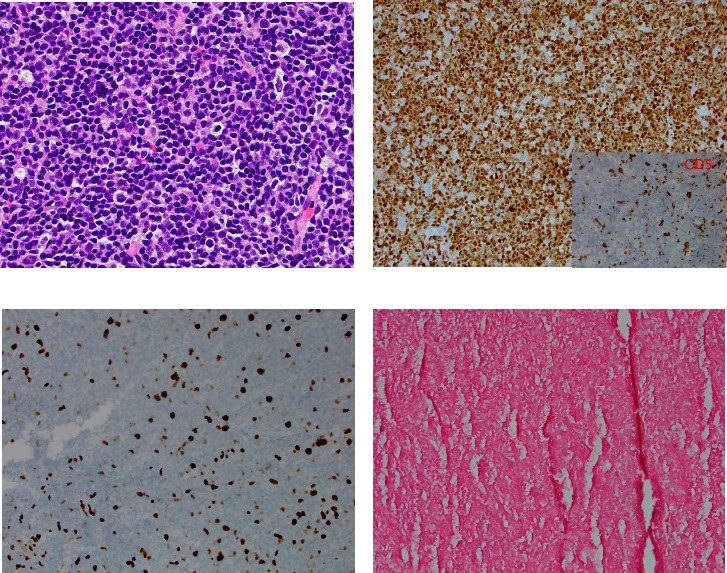
CD5-negative, EBV-negative low-grade MCL on cecal biopsy in November 2021. (a) Diffuse monotonous small, atypical lymphocytes with slightly irregular nuclear contour (hematoxylin-eosin, original magnification ×200). (b) The atypical lymphocytes exhibit strong expression of CD20 and pax-5 (not shown), diffuse cyclin D1 positivity (1B), and lack of CD5 expression (1B inset). (c) Immunohistochemical stain for Ki-67 revealed a low proliferation index of approximately 10%. (d) EBER in situ hybridization for EBV showed no EBV infection.

The patient came to our hospital for care of lymphoma in May of 2020. Disease progression was noted in April of 2021 with a large cecal mass identified through colonoscopy. A CT scan showed asymmetric thickening of the cecum and an increase in spleen size to approximately 24 cm. A cecum biopsy in November 2021 (Figure 1) showed persistent classical mantle cell lymphoma. The lymphoma cells were mostly small (1A). Cyclin D1 was positive, while CD5 and SOX11 were negative in the lymphoma cells (1B). The proliferation index (Ki-67) was estimated to be 10% (1C). In situ hybridization (ISH) was negative for EBER (1D). He was treated with weekly Rituxan x 4 and Revlimid from May 2021 to April 2022. He had evidence of persistent disease by imaging, low-grade fever, and a low white blood cell count (2.78 × 10^9^/*µ*L) after Revlimid therapy. Serum EBV and CMV levels determined by PCR were negative. Zanubrutinib was started in June 2022, and the patient achieved a very good partial response after 3 months of therapy by imaging.

**Figure 2:**
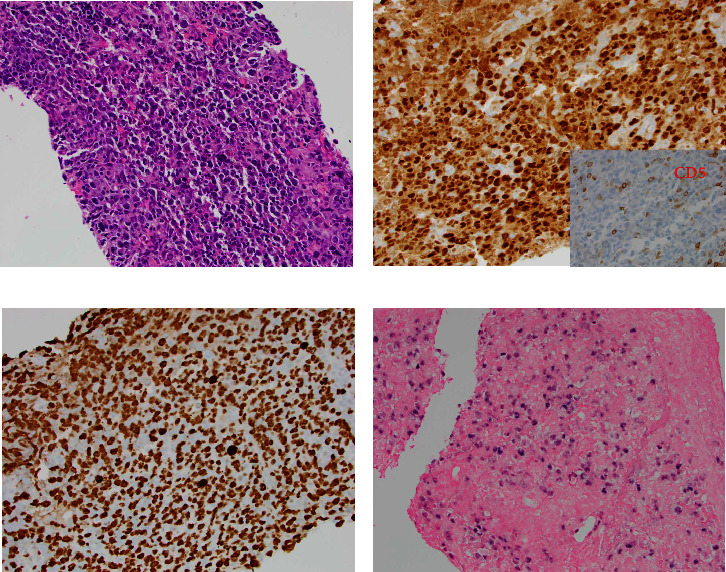
Left axillary lymph node biopsy of May 2023 showing high grade pleomorphic mantel cell lymphoma. (a) H&E staining at 20x magnification showing large, atypical lymphocytes with fine chromatin in the background of necrosis. (b) Immunohistochemical staining for cyclin D1 was diffusely positive, while CD5 was negative (insert). (c) Immunohistochemical stain for Ki-67 revealed a high proliferation index (∼90%) and large nuclear size. (d) EBER in situ hybridization for EBV detected diffusely positive cells.

**Figure 3:**
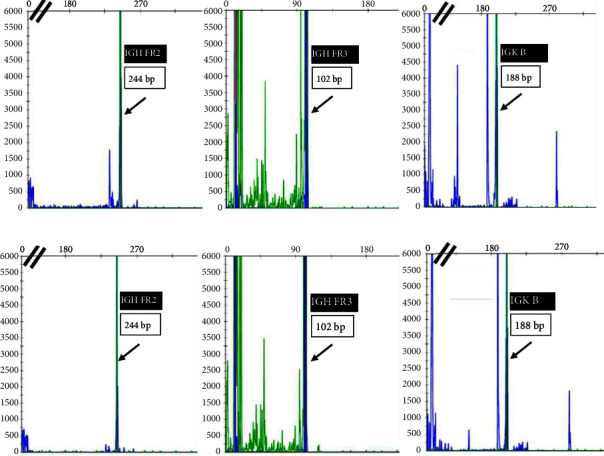
B-cell gene rearrangements in the specimen of November 2021 (a) and May 2023 (b) showing the exact same clonal peaks, indicating evolution from the same clone.

He presented with recurrent fevers in May of 2023. An infectious work-up revealed a serum EBV titer of 803,517 IU/ml. An imaging study showed increased lymphadenopathy. Biopsy of the left axilla lymph node (Figure 2) showed transformed pleomorphic variant of mantle cell lymphoma. The lymphoma cells were mostly large with irregular nuclear contours, frequent mitotic figures, and apoptotic bodies (2A). Again, cyclin D1 was positive, CD5 and SOX11 were negative in the lymphoma cells (2B). CD5 negativity was also confirmed by flow cytometry. Ki-67 was estimated at 90% (2C) and many of the large cells were positive for EBER-ISH (2D). The lymphoma cells were strongly positive for P53 stain, suggestive of TP53 mutation. FISH for t(11,14) IgH::CCND1 was positive (not shown), confirming the diagnosis of MCL. No rearrangements in BCL2, BCL6, or MYC were detected. PCR analysis revealed that the sizes of the clonal peaks in immunoglobulin heavy chain and light chain gene rearrangements were identical to those observed in cecum biopsy conducted in November 2021 (Figure 3), confirming their same clonality.

The patient was treated with obinutuzumab and polatuzumab for EBV infection. His serum EBV titer improved significantly and the EBV titer in September 2023 was 11027 IU/ml. He received Tecartus CAR-T cell infusion in September 2023 and the most recent PET scan in December 2023 showed no lymphadenopathy. The serum EBV titer was also negative.

## 3. Discussion

MCL is a non-Hodgkin lymphoma characterized by the t(11; 14) (q13; q32) translocation, resulting in the overexpression of cyclin D1 [8]. MCL exhibits a diverse range of presentations, ranging from indolent courses with slow course to aggressive types with rapid progression or as something in between [9]. It is categorized into low- and high-grade variants. The low-grade classical lymphocytic variant tends to progress more slowly. In contrast, high-grade subtypes, such as the pleomorphic and blastoid variants, typically advance more rapidly and exhibit a higher prevalence of molecular and cytogenetic abnormalities than the low-grade subtypes [9].

EBV infections are more often associated with high grade B-cell lymphoma, such as classical Hodgkin lymphoma, diffuse large B-cell lymphoma, Burkitt lymphoma, and less frequently with low-grade lymphomas, such as follicular lymphoma, marginal zone lymphoma, and chronic lymphocytic leukemia (CLL) [10]. Generally, patients with EBV + lymphomas have worse prognoses than those with EBV negative lymphomas. Patients with EBV + classical Hodgkin lymphoma had worse three-year event-free survival and overall survival compared to EBV negative patients with classical Hodgkin lymphoma [11]. Another study found that EBV infectivity in diffuse large B-cell lymphoma patients was associated with older age and a more advanced disease state [12]. Compared to EBV negative patients with CLL, EBV + patients with CLL have a shorter response time to treatment and lower overall survival rates [13]. Baek et al. also found that cell-free serum EBV DNA at the time of diagnosis was associated with more advanced disease, higher disease relapse, and inferior survival [14].

Among EBV + non-Hodgkin lymphomas, MCL represents only 11.1% of the cases [15]. Although MCL is not usually considered an EBV + lymphoma [16], EBV can infect MCL in rare cases. Among patients with MCL, the incidence of EBV + MCL is low, with one report reporting an infection rate of 5.8% [6]. Interestingly, this study found that clinical and pathological findings were comparable between EBV+ and EBV-negative MCL patients. In the majority of EBV + MCL cases, EBV infected the background cells rather than the tumor cells. The study further suggested that MCL tumor cells may exhibit resistance to EBV infection. On the other hand, in patients with MCL where EBV DNA was detected in the peripheral blood, both progression-free survival and overall survival were lower compared to those without EBV infection [16]. Additionally, one study reported a case of an individual with a history of hypersensitivity to mosquito bites who developed EBV + MCL. The authors of this study hypothesized that EBV may have contributed to the development of MCL in this patient [17].

The aggressive transformation of a low-grade MCL to a high-grade MCL may present with characteristic molecular and cytogenetic findings. Genetic changes that may drive the transformation of MCL include MYC rearrangements, NOTCH2 mutations [18], and KMT2B mutations [19]. A previous study revealed that patients with high Ki-67 levels (≥50%) had specific mutations and worse survival than those with lower Ki-67 levels (<50%) [19].

The effect of EBV infection on MCL disease progression is unclear. Kanail et al. reported a 70‐year‐old man with MCL who relapsed with a form of nodal lymphoma best interpreted as the combination of MCL and classic Hodgkin lymphoma 9 years after autologous peripheral blood stem cell transplantation [20]. Two separate areas were present in that patient's specimen, one area being typical MCL that was CD5+, Cyclin D1+, and EBV-, while the other area being classical Hodgkin lymphoma like that was CD30+, CD15+, EBV+, CD5-, and cyclin D1-. Both areas showed t (11; 14) rearrangement and same B-cell gene rearrangement, suggesting that EBV infection in the context of MCL can lead to transformation into a related disease. There is only one case report describing high grade pleomorphic transformation of CD5+ MCL from EBV infection after a clinical course of 19 years [7]. Higuchi et al. described blastoid variant of mantel cell lymphoma in a patient with current infectious mononucleosis-like symptoms [21], while Terasawa et al. reported a case of EBV-associated transformation of MCL into diffuse large B-cell lymphoma [22]. In our case report, we described another patient with classic but CD5-negative MCL that progressed to aggressive pleomorphic variant after EBV infection. SOX11 was negative in our case. If it is positive, it can help to distinguish MCL from cyclin D1 positive diffuse large B-cell lymphoma as reported by Hsiao et al. [23]. CD5-negative MCL has been reported to be associated with better prognosis than CD5+ MCL [4]. The patient was EBV negative when the diagnosis was a low-grade lymphocytic variant but became EBV positive when the diagnosis progressed to a high-grade pleomorphic variant. Gene rearrangement studies in both specimens showed exactly the same peaks, confirming their common clonal origin.

The treatment for MCL includes conventional chemoimmunotherapy, stem cell transplantation, BTK, Bcl2, and ROR1 inhibitors, as well as anti-CD19 chimeric antigen receptor therapy (CAR-T), and recently developed bispecific antibodies against CD19 and CD20 [24]. The recognition of the role of EBV infection in MCL and its transformation raises the need to include EBV infection detection and control in MCL management.

In summary, we reported a case of EBV positive or possible EBV driven transformation of CD5-negative MCL from a lower grade variant to a more aggressive pleomorphic variant. Therefore, we advise practitioners to check for EBV infection in patients with MCL transformation for better treatment and patient management.
